# Weight loss prior to diagnosis of first-episode psychosis predicts subsequent weight gain: a retrospective cohort study of the UK Clinical Practice Research Datalink (CPRD) primary care database

**DOI:** 10.1136/bmjment-2026-302720

**Published:** 2026-07-16

**Authors:** Brian O’Mahony, Richard I G Holt, Emanuele F Osimo, Karla V.B Hitchins, Brian O’Donoghue, Benjamin I Perry

**Affiliations:** 1 Psychosis Research Centre, University College Dublin, Dublin, Ireland; 2 Institute for Mental Health, University of Birmingham, Birmingham, UK; 3 Human Development and Health, Faculty of Medicine, University of Southampton, Southampton, England, UK; 4 Psychiatry, University of Cambridge, Cambridge, UK; 5 Birmingham and Solihull Mental Health NHS Foundation Trust, Birmingham, UK

**Keywords:** Psychotic Disorders, Schizophrenia

## Abstract

**Background:**

Post-psychosis weight gain is commonly rapid, yet prediagnosis weight changes are under-researched. The untreated phase of first-episode psychosis (FEP) can be characterised by behaviour which could lead to weight loss, triggering physiological adaptations which prime the body to regain weight.

**Objective:**

We aimed to investigate the prevalence and extent of weight loss prior to diagnosis of FEP, and to investigate its impact on subsequent weight gain.

**Methods:**

We used Clinical Practice Research Datalink primary care data, supplemented with linked hospital episode statistics, ethnicity and deprivation data to estimate pre-FEP weight loss prevalence. We then used multiple linear regression to assess its association with subsequent weight change, while adjusting for relevant confounders.

**Findings:**

Primary analysis included 369 individuals with adequate data. 53% of individuals experienced weight loss before FEP. Using percentage weight change as an outcome, individuals with pre-FEP weight loss gained weight post-FEP at over twice the rate of those without prior loss (8.93% (95% CI 6.90% to 10.96%) bodyweight per year vs 4.07% per year (95% CI 1.90% to 6.24%); p=0.005). Each 1% of pre-FEP weight loss predicted 0.41% (95% CI 0.23% to 0.59%) additional annual weight gain (p=0.004), most pronounced within 6 months and independent of antipsychotic treatment selection. Using kilogram per year as an outcome, average gain (6.49 kg (95% CI 5.28 kg to 7.70 kg)) exceeded average prior loss (4.83 kg (95% CI 4.00 kg to 5.66 kg)) by 1 year. Findings were robust across sensitivity analyses.

**Conclusions:**

Pre-FEP weight loss is common and predicts rapid post-diagnosis gain. While all patients were expected to gain weight, those with pre-FEP weight loss gained double the weight of those who had not lost weight.

**Clinical implications:**

Clinicians treating individuals with FEP should enquire about weight loss in the pretreatment period, which predicts extra weight gain in the initial period following diagnosis. This could inform early intervention to avoid consequences of short-term weight gain.

**WHAT IS ALREADY KNOWN ON THIS TOPIC**

- Weight gain is commonly rapid in first-episode psychosis (FEP) and is a leading contributor to the decreased life expectancy of this patient group. The untreated phase of FEP is often characterised by withdrawal, disorganisation and poor self-care.

**WHAT THIS STUDY ADDS**

- We show that weight loss is common in the pretreatment phase of FEP, and that those who experienced pretreatment weight loss can expect to gain double the rate of those who did not lose weight.

**HOW THIS STUDY MIGHT AFFECT RESEARCH, PRACTICE OR POLICY**

- Clinicians treating individuals with FEP should routinely inquire about weight changes prior to the individual’s first presentation. Doing so might inform the need for more intensive cardiometabolic monitoring and intervention.

## Introduction

 First-episode psychosis (FEP) is typically associated with rapid weight gain, particularly in the first few months of treatment.^1^ However, physical health changes may also occur during the untreated period, preceding secondary mental health presentation. Untreated FEP may be characterised by disorganisation, poor self-care and social isolation,^2^ which may alter diet and physical activity. These factors may contribute to weight loss prior to treatment initiation.

Historical clinical descriptions support the possibility of weight loss during acute psychosis. Kraepelin, writing in the premedication era, proposed that weight loss in the acute stages of psychosis reflected systemic illness, and that subsequent weight restoration, alongside improved clarity of thought, indicated remission.^3^ The relationship between symptomatic improvement and weight change remains debated,^4^ and contemporary interpretation is complicated by the established cardiometabolic effects of antipsychotic medication. Antipsychotics can induce weight gain and metabolic disturbance in both healthy individuals and those with psychosis.^5 6^ Notably, weight gain following antipsychotic initiation is more pronounced in FEP than in chronic psychosis.^1 6 7^

The differential susceptibility to weight gain by illness chronicity has been attributed to younger age, lower baseline weight, underlying metabolic vulnerability and a plateauing of antipsychotic-associated weight gain over time.^8^ An additional, and less explored, explanation is that a component of post-treatment weight gain reflects physiological rebound following weight loss prior to treatment. Weight loss is associated with adipose tissue stress and inflammatory changes, endocrine adaptations, and reductions in resting metabolic rate.^9 10^ These responses favour weight restoration and are consistent with a high probability of weight regain after weight loss.^11^

Existing research of weight trajectories in psychotic disorders has predominantly recruited individuals who have already received an FEP diagnosis and therefore cannot characterise weight change preceding first clinical presentation. Studies of clinical high risk of psychosis populations are constrained by low FEP transition rates; uncertain representativeness; and limited availability of repeated physical health measures.^12^

Routine primary care health record datasets offer a compelling opportunity to address these limitations. These datasets potentially capture longitudinal clinical information across the lifetime. Primary care health utilisation may increase during the prodromal period as well as following FEP.^13^ We therefore used a large routine anonymised primary care health record database to characterise weight trajectories before FEP and examine associations of pre-FEP weight change with subsequent weight gain after FEP.

## Methods

### Design

This was a retrospective cohort study using routinely collected primary care data. We used the large Clinical Practice Research Datalink Gold electronic database. We used data from English practices due to the availability of Townsend deprivation scores, alongside linkage with Hospital Episode Statistics (HES) and Office for National Statistics records.

### Study population

Our sample included individuals aged 16–35 years who received a first recorded diagnosis of a psychosis-spectrum disorder between 1 January 2005 and 31 December 2015, from primary care records or HES, with the study period ending on 31 December 2020. We included individuals who would be eligible for care within Early Intervention Services in England, broadly comprising individuals experiencing psychotic symptoms for the first time (International Classification of Diseases (ICD) codes: F06.0–2, F20–F31, F32.3, F33.3, F53.1). We included individuals with at least three weight recordings within 3 years before and 18 months after FEP.

### Exposures

Weight measurements are not systematically recorded in routine healthcare. We therefore operationalised pre-FEP weight change using multiple ascertainment windows to assess robustness to irregular weight recording and to explore the impact of alternative exposure definitions.

In the primary analysis (‘strict definition’), we defined pre-FEP weight change as the difference between (1) the individual’s maximum recorded weight prior to FEP and (2) an index weight, defined as the weight measurement closest in time to the recorded FEP diagnosis date and occurring within the 6 months before, or 1 month after, diagnosis of FEP. We selected the 6-month window to account for potential delays in the recording of FEP in primary care records, and to ensure a weight that could be reasonably expected to reflect the individual’s true weight at FEP. To do this, we plotted the rates of weight change for each individual and found that rates of weight change followed a normal distribution with mean close to zero for each 2-month period prior to diagnosis (0–2 months, 2–4 months and 4–6 months). Conversely, the time between the index date and the first month was highly skewed (skewness=2.71 and kurtosis=8.94), meaning that weights beyond 1 month following diagnosis would not be accurate proxies. Online supplemental table 2 provides a numerical summary of the distribution of individuals’ weight changes, split by time from the index diagnosis.

We reasoned that, at minimum, at least three weights would be required to assess the two weight trajectories (before and after FEP), which considerably reduced the included sample. We chose to prioritise internal validity at the expense of sample size for our primary analysis, because the strict definition permitted an approximation of weight at the time of diagnosis of FEP, as well as sufficient weight measurements before and after diagnosis.

We relaxed the criteria in secondary analysis to increase sample size. First, under the ‘*extended definition*’, index weight was defined as the last recorded weight before the recorded FEP diagnosis date (ie, without restricting the measurement to a 6-month window). Second, under the ‘*loose definition*’, index weight was defined as the closest recorded weight to the FEP recorded diagnosis, whether this occurred before or after the recorded diagnosis.

**Figure 1:**
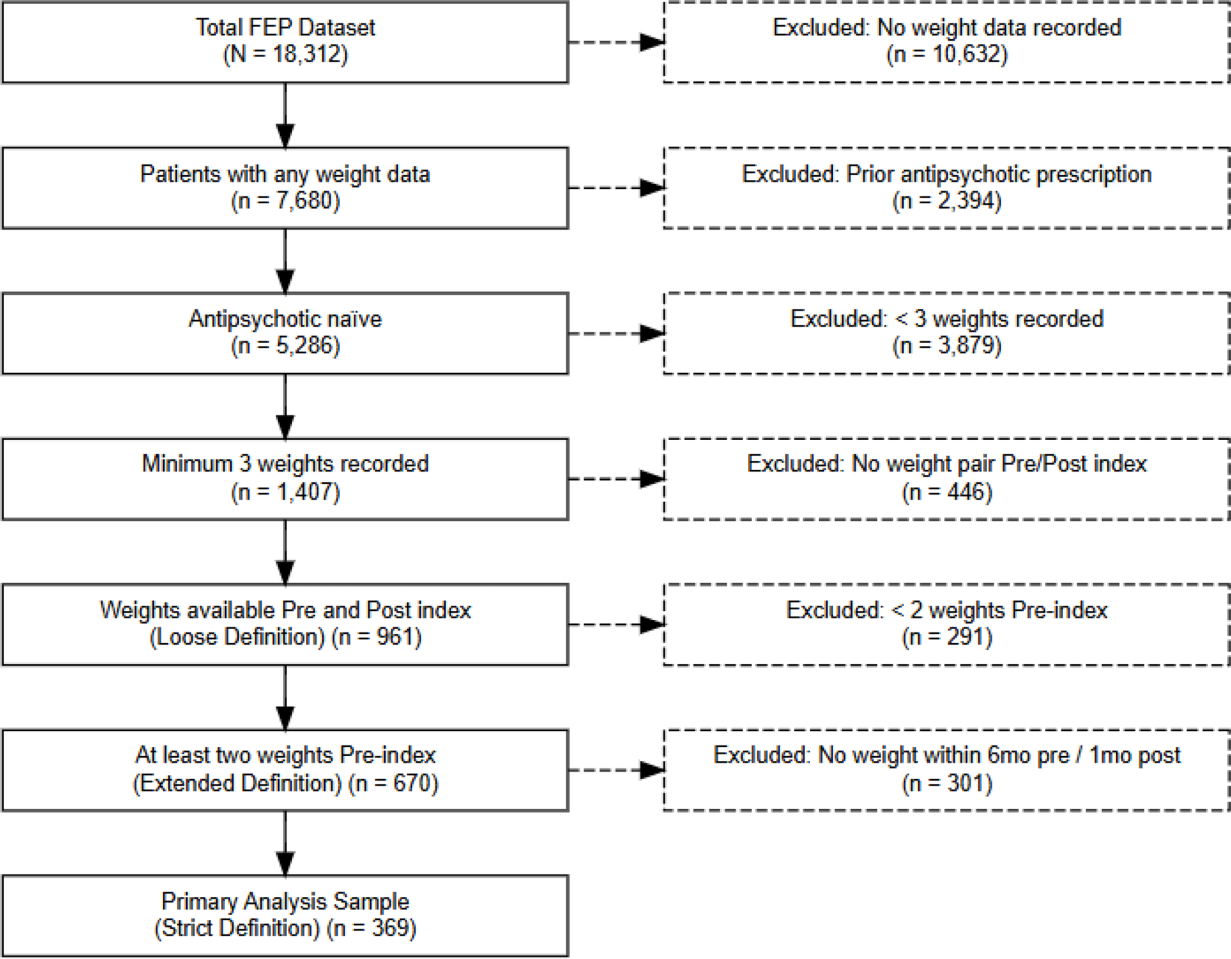
Flowchart of number of people in the CPRD dataset given different sets of inclusion criteria. Note: All included participants had eligibility for linkage across included data sources (primary care, HES APC and OP, alongside ethnicity ascertainment and practice-level Townsend score). APC, admitted patient care; CPRD, Clinical Practice Research Datalink; FEP, first-episode psychosis; HES, hospital episode statistics; mo, months; OP, outpatient.

The *extended definition* sample included individuals whose ‘index’ weight, that is, the inflection point in a line made up of three datapoints, could have occurred before FEP in some instances.^2^ The *loose definition* allowed for the inflection point to occur at any time in the peri-FEP period, potentially after FEP in some cases. This was considered a less favourable definition given the evidence of the rapidity of weight gain following FEP,^1^ and a similar indication of rapid weight gain in our data. Figure 1 shows a flowchart of the inclusion criteria for each sample.

For all definitions, weight change was characterised in two ways to explore consistency: (1) as a binary indicator (any weight loss vs no weight loss) and (2) as a continuous measure (percentage change from maximum pre-FEP weight).

### Outcome

Our primary outcome was percentage of weight change per year from the individual’s index weight, using a time × percentage weight change interaction term, thus effect estimates represent the effect of pre-FEP weight loss on their rate of post-FEP weight gain. We chose percentage weight change over change in percentage body mass index (BMI) because they are mathematically equivalent, while percentage weight is easier to interpret and is used in major obesity trials and recommended in European Medicines Agency and Food and Drug Administration guidance. We chose percentage weight change over change in absolute BMI because absolute BMI change does not convey proportional change without context; for example, a –1 kg/m² change carries different meaning if the baseline BMI is 22 kg/m² compared with a baseline BMI of 40 kg/m².

### Covariates

We constructed a Directed Acyclic Graph a priori to inform covariate selection (online supplemental figure 1). After aligning to available data, we selected age, sex, BMI at the index date, diagnosis, socioeconomic status, ethnicity and location. We used age as a continuous covariate as we considered any categorical separation of the 16–35 year-olds in the dataset would have been arbitrary. We operationalised the antipsychotic covariate as the first recorded antipsychotic prescription, having filtered out any individual with a recorded prescription of an antipsychotic greater than 1 month before their index diagnosis. For socioeconomic status, we used the Townsend Index, whose scores for analysis were based on the 2011 census, an approximate mid-point of our study timeframe. We also included number of general practitioner visits which occurred during the study period (3 years before and 18 months after the index record) as a proxy for medical unwellness. We included all of these listed variables as potential confounders for adjusted analysis.

### Statistical analysis

We used linear mixed-effects models to estimate the longitudinal trajectories of weight change across the different drug and time factors. This allowed for the model to use general trends that apply to the whole group, along with individual variations (random effects) to provide a more accurate picture of how weight changes across the sample. We modelled time and drug factors as fixed effects, which represent the specific factors the model is investigating. We simultaneously accounted for within-subject correlation and baseline variability through random intercepts. This accounts for the fact that every person begins the study with a different baseline weight, allowing for rate of change to be estimated independent of index weight.

We estimated the average annual rate of percentage weight change by investigating the slopes of the interaction terms using the emmeans package for R.^14^ An interaction term multiplies two variables together in the linear model, allowing for interpretation of a shared effect, in this case weight change per year. We used pairwise contrasts to assess the differences in slopes between the two groups (weight loss vs no weight loss). Pairwise contrasts allowed us to statistically compare the results of two different groups (those who lost weight vs those who did not) side-by-side. While the interaction term informed us of the weight gain happening per year, the pairwise contrast specifically tests the gap between the two groups. We used the lme4 R package to fit the models,^15^ using the BObyQA optimiser to facilitate convergence (online supplemental methods). The optimiser allows the model to find the best fit for the data when calculations are complex. We conducted multiple imputation using chained equations (20 imputations) to impute missing covariate data using the MICE package for R,^16^ that is, we did not impute weights (online supplemental methods). Online supplemental table 1 shows the auxiliary variables used in the MICE (Multivariate Imputation by Chained Equations) process. We performed two-sided tests and set the α level at 0.05.

We conducted several sensitivity analyses. First, we additionally adjusted for the time interval between the individual’s maximum recorded weight and the index weight measurement to ensure that the dependent variable (weight loss) was not solely due to the individual having had a prolonged time period in which they may have lost weight. In a separate model, we adjusted for sociodemographic covariates only to assess for overadjustment and to establish the differential confounding impact of sociodemographic versus clinical or medication-related factors. Third, we assessed whether results were sensitive to how weight change was parameterised by replacing percentage weight change with (1) absolute weight change and (2) the rate of weight change. We felt percentage weight change was more suitable than absolute weight change, which would have different effects on individuals of different weight. We assessed the effect of rate of weight change to assess if weight regain differed between individuals who rapidly lost weight pre-FEP, rather than the amount of weight loss being key. Fourth, we examined potential non-linearity by modelling weight change using natural cubic splines in both unadjusted and adjusted models, which we examined due to the well-described non-linearity of weight gain following treatment of psychosis. Fifth, we conducted a post-hoc exploratory analysis of our primary analysis stratified by sex, although these analyses lacked power to effectively determine between-group differences. Finally, we fitted a model with the intercept constrained to zero, thereby centring weight change on the index weight. Although this constrains the model, and thus increases its sum of squared errors, it ensures that each individual has a ‘weight gain’ of zero at their index episode, rather than the model, for example, allowing individuals to have a ‘weight gain’ of 1 kg at their index episode, which does not make sense clinically.

## Results

### Study population

Our sample frame included 18 312 individuals with first recorded psychosis spectrum disorder between 2005 and 2015. Our primary analysis (*strict definition*) included 369 individuals (mean age 26.02 years (SD=5.98); male sex 124 (33.6%); 204 (55.3%) of whom were of white background (individuals classified as either white British, white Irish or any other white background). Missing sample analysis showed differences between the included and excluded samples. The included samples had slightly higher ethnic diversity, were more likely to be female, and were slightly younger (see online supplemental table 3). Secondary analyses included 670 for the *extended index* group (those with at least two weight measurements prior to FEP) and 961 for the *loose definition* (those with at least three weights, with one weight before and after FEP). Table 1 reports the characteristics of each of these groups. Included individuals had a median of 5 weights performed (IQR 3–7) between 2002 and 2020.

**Table 1:** Characteristics of the three samples used for analysis

Characteristic	Strict definition (N=369)	Extended definition (N=670)	Loosest definition (N=961)
Female (%)	245 (66.4)	476 (71.0)	633 (65.9)
Age at FEP onset	26.02 (5.98)	26.25 (5.84)	26.15 (5.81)
Diagnosis	N (%)	N (%)	N (%)
Acute and transient psychotic disorder	21 (5.7)	36 (5.4)	48 (5.0)
Delusional disorder	10	17 (2.5)	18 (1.9)
Depression with psychotic symptoms	44 (11.9)	80 (11.9)	109 (11.3)
Mania with psychotic symptoms	78 (21.1)	158 (23.6)	212 (22.1)
Organic psychotic disorder	*	*	*
Postpartum psychosis	10	16 (2.4)	19 (2.0)
Schizoaffective disorder	10	18 (2.7)	22 (2.3)
Schizoid/schizotypal personality disorder	*	*	*
Schizophrenia	19 (5.1)	39 (5.8)	67 (7.0)
Substance-induced psychotic disorder	10	10	15 (1.6)
Unclassified/other psychotic disorder	177 (48.0)	286 (42.7)	436 (45.4)
Missing	0 (0.0)	1 (0.1)	3 (0.3)
Location	N (%)	N (%)	N (%)
East Midlands	10	15 (2.2)	18 (1.9)
East of England	22 (6.0)	57 (8.5)	82 (8.5)
London	61 (16.5)	113 (16.9)	183 (19.0)
Northeast	10	23 (3.4)	30 (3.1)
Northwest	83 (22.5)	135 (20.1)	182 (18.9)
Southeast	94 (25.5)	164 (24.5)	228 (23.7)
Southwest	27 (7.3)	55 (8.2)	76 (7.9)
West Midlands	32 (8.7)	59 (8.8)	78 (8.1)
Yorkshire and The Humber	*	15	23 (2.4)
Missing	23 (6.2)	36 (5.4)	61 (6.3)
Ethnicity	N (%)	N (%)	N (%)
Black African or Caribbean	33 (8.9)	54 (8.1)	84 (8.7)
East Asian	10	10	16 (1.7)
Mixed	95 (25.7)	176 (26.3)	244 (25.4)
Other	10	15	22 (2.3)
South Asian	20 (5.4)	35 (5.2)	59 (6.1)
Any white background	204 (55.3)	380 (56.7)	536 (55.8)
Townsend Quintile	N (%)	N (%)	N (%)
1	31 (8.4)	63 (9.4)	84 (8.7)
2	44 (11.9)	94 (14.0)	128 (13.3)
3	66 (17.9)	122 (18.2)	180 (18.7)
4	86 (23.3)	172 (25.7)	249 (25.9)
5	142 (38.5)	219 (32.7)	320 (33.3)
Baseline BMI			
Mean (SD)	25.73 (6.84)	25.97 (6.68)	25.83 (6.46)
Missing	14 (3.8)	42 (6.3)	85 (8.8)
Recorded antipsychotic prescription	236 (64)	397 (59.3)	596 (62.0)

Cells with counts less than 15 have been altered in conjunction with National Health Service digital reporting guidelines.

BMI, body mass index; FEP, first-episode psychosis.

### Primary analysis

**Figure 2:**
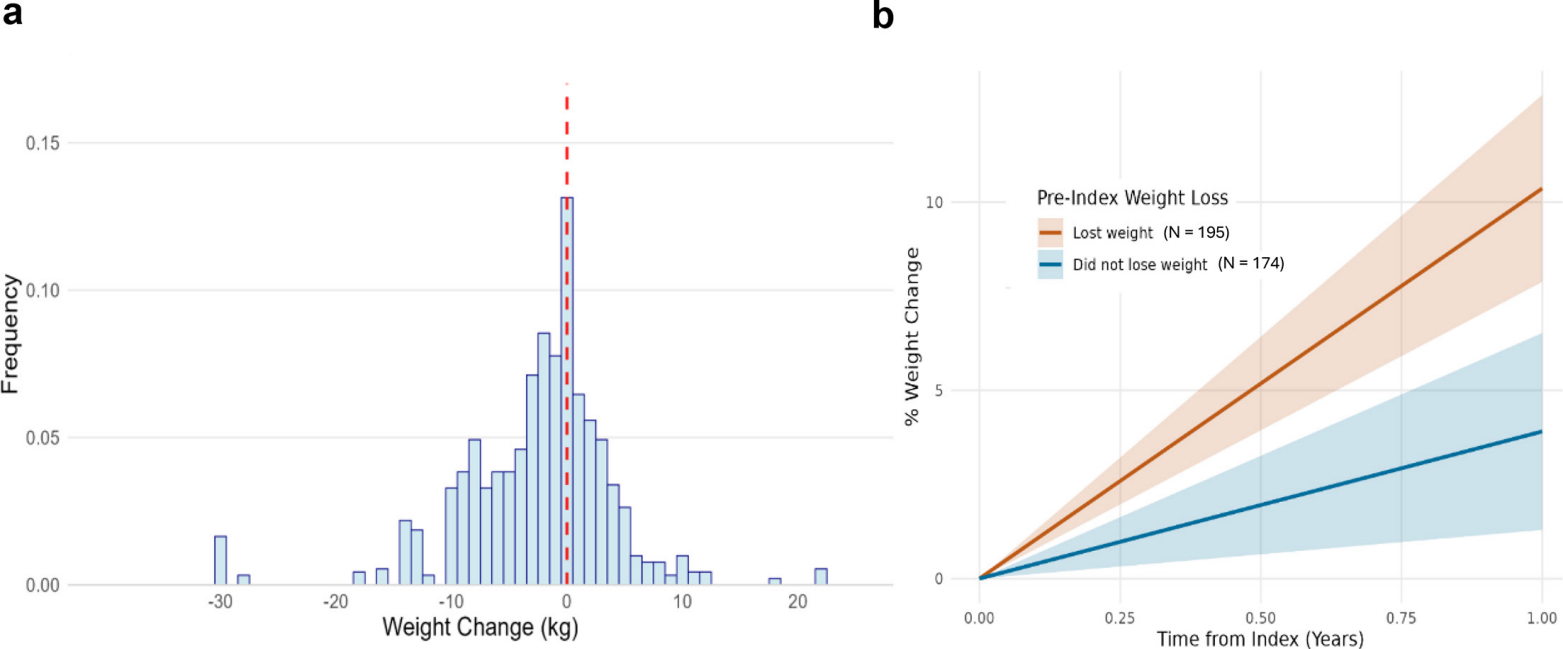
Weight changes before (a) and after (b) diagnosis of first-episode psychosis in the primary analysis cohort (n=369).

From the *strict definition* cohort, 195 (53%) lost weight prior to diagnosis of FEP. Of those who lost weight, the mean weight loss was 4.63 kg (95% CI 4.21 kg to 5.05 kg, range 0.2 kg to 25 kg). In the model using weight loss as a binary variable, those who lost weight were expected to gain 8.93% per year (95% CI 6.9% to 10.96%) of their body weight versus 4.07% per year (95% CI 1.9% to 6.24%) in those who did not lose weight, a difference of 4.86% per year (95% CI 1.92% to 7.80%, p=0.001). The results were robust to covariate adjustment including antipsychotic choice (4.15% difference in weight gain per year (95% CI 1.10% to 7.22%, p=0.005)). Figure 2 shows the distribution of weight changes prior to diagnosis, as well as the expected weight gain in the first year of those who had lost weight versus those who had not lost weight, under the zero-intercept model. These results were consistent and robust across all sensitivity analyses, using different models, covariates and samples (online supplemental table 4).

Defining pre-FEP weight loss as a continuous variable, every 1% loss in body weight was associated with a 0.41% (95% CI 0.23% to 0.59%) extra weight gain per year (p<0.001). The results were robust to covariate adjustment including antipsychotic choice (0.32%) extra weight gained per year (95% CI 0.14% to 0.49%, p<0.001). These results were consistent and robust across all sensitivity analyses (online supplemental table 4). A summary of the primary analyses is shown in the online supplemental table 5.

### Secondary analyses

The effect of pre-index weight loss on post-index weight gain was larger over time periods closer to the index date. Every 1% of weight loss prior to diagnosis was expected to lead to an extra 0.47% weight gain per year (95% CI 0.25% to 0.69%) in the following 12 months in the unadjusted model, and 0.48% per year in the adjusted model (95% CI 0.27 to 0.69%). Over the course of the first 6 months this rate of extra weight gain increased to an extra 0.62% weight gain per year (95% CI 0.35% to 0.89%) in the unadjusted model, and 0.60% per year (95% CI 0.32% to 0.88%) in the adjusted model.

**Figure 3:**
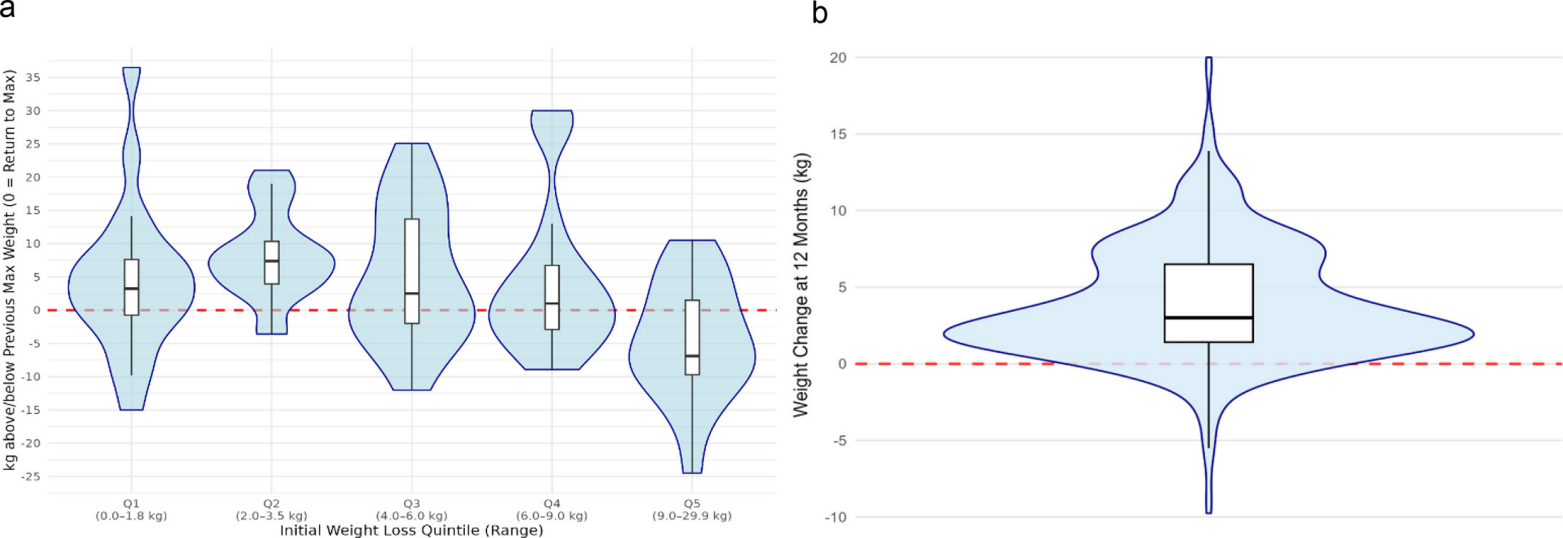
Weight changes following diagnosis. (a) Those who had lost weight prediagnosis, with each violin representing a quintile based on weight loss prediagnosis. (b) Those who did not lose weight prediagnosis.

Using the individual’s first weight at least 1 year following diagnosis, this cohort had gained an average of 6.49 kg (95% CI 5.28 kg to 7.7 kg), having lost an average of 4.83 kg (95% CI 4 kg to 5.66 kg). Of this sample of 98 individuals, 64 (65.3%) had gained more weight than they had lost. Figure 3 shows the amount of weight gained in the first year after diagnosis, compared with their previous maximum weight, where the width of the ‘violin’ represents the frequency of data points at that value; wider sections indicating higher data density.

## Discussion

This is among the first studies to examine weight trajectories of individuals with FEP including the period before first recorded FEP diagnosis. We found that weight loss before FEP diagnosis is common and a strong risk factor for rapid post-FEP weight gain that exceeds pre-FEP weight loss, independent of antipsychotic choice. Most individuals in our sample gained weight after FEP, however, those who lost weight before FEP gained weight at approximately double the rate compared with those who did not. Finally, we also found that this additional weight gain was more pronounced in the early stages of treatment. Our findings were robust across sensitivity analyses including covariate adjustment.

Prediagnosis weight loss was associated with substantially greater weight gain during the treated period following FEP. Across our analyses, individuals with evidence of pre-FEP weight loss were estimated to gain a twofold higher amount of body weight than those without evidence of pre-FEP weight loss. This pattern is consistent with the presence of compensatory weight-restoration mechanisms after weight loss and may help explain inter-individual heterogeneity in post-FEP weight trajectories. Thresholds used to define ‘clinically significant’ weight gain vary across guidelines and studies (typically 3–10%).^17 18^ Our results suggest that prediagnosis weight loss may shift a meaningful proportion of individuals across commonly used action thresholds. This has potential implications for targeting preventive interventions in early psychosis services, where eligibility for lifestyle and pharmacological strategies is often operationalised using weight-change criteria.^17 19^ Beyond eligibility for additional treatment, short-term weight gain carries consequences for the individual such as stigma and shame. These damaging psychological burdens may then be further compounded by self-cessation of the antipsychotic medication contributing to this weight gain, as well as psychiatric hospitalisation. As such, clinicians aware of the individual’s prediagnosis weight trajectory, and its impact on postdiagnosis weight gain, would likely make more informed and collaborative decisions with the individual. Doing so would therefore aid in avoiding the shame, stigma, hospitalisation, treatment non-adherence and loss of quality of life all associated with antipsychotic induced weight gain.^20^

We found a ‘dose-dependent’ effect of pre-FEP weight loss on subsequent weight gain, aligning with evidence that physiological adaptations to weight loss scale with the amount of weight lost.^21^ Consistent with this interpretation, using the rate of prediagnosis weight change as the exposure yielded similar inferences, suggesting that the quantity of weight loss may be the key determinant of the subsequent trajectory. Our results are therefore more consistent with models of defended or individualised weight regulation, where trajectories are shaped by prior position relative to an individual’s ‘settling point’ or defended range, than with models positing self-reinforcing, runaway weight gain driven by positive feedback processes.^22^

We found that a substantial proportion of individuals with pre-FEP weight loss had regained weight exceeding that which they lost. The trajectory of weight gain after antipsychotic initiation is well described as non-linear, with a steep early increase followed by tapering.^1^ One interpretation is that antipsychotics contribute to an upward shift in the defended weight, leading to rapid initial gain that slows as weight stabilises around a higher level. Together, these findings suggest that prediagnosis weight trajectories may be informative for anticipating the magnitude and timing of weight gain following treatment initiation.

There was limited attenuation of effects after adjustment for measured covariates, including recorded antipsychotic prescription. This means that while antipsychotic use strongly correlates with weight gain, it did not markedly impact the *additional* weight gain of someone who lost weight prior to FEP, compared with someone who did not lose weight. Although antipsychotic exposure is an established contributor to weight gain following FEP, weight gain after antipsychotic exposure varies substantially between individuals. Our findings indicate that pre-FEP weight trajectories may account for some of this heterogeneity and may provide prognostic information beyond routinely recorded treatment variables, and may have relevance for consideration in population-specific prognostic tools.^23^ Future studies with more complete medication and dosing data should test whether antipsychotic-associated weight gain among individuals with stable pre-FEP weight is similar in magnitude to the ‘excess’ gain seen in those with pre-FEP weight loss. Additionally, future studies with larger sample sizes should seek to consider sociodemographic or diagnostic subgroup analysis to enable more accurate and/or targeted clinical recommendations for weight gain prevention.

There remains ongoing work to determine the optimal preventative interventions for weight gain in FEP. Metformin has been suggested for use alongside some instances of antipsychotic initiation,^17^ but the overall evidence of pharmacological intervention in drug-naïve, first episode psychosis is limited.^24^ Other options include behavioural interventions, including physical activity.^25^ Exercise increases lipolysis and reduces inflammation, which are key factors in weight regain.^26^ While psychosis does not preclude someone from engaging in and benefitting from physical activity, biopsychological sociodemographic challenges for many individuals with FEP may complicate uptake and adherence to health behavioural interventions.^27 28^ Additionally, unhealthy food environments are closely linked to weight gain.^29^ As such, cardiometabolic health should be tailored to the life and environment of the individual, who should be offered holistic interventions which improve the environment, alongside any behavioural or pharmacological interventions.

Our study has a number of limitations. First, our strict inclusion criteria, while improving face validity by confirming that weight changes were temporally linked to the FEP diagnosis, limited the available analytic sample to 2% of the sample frame and increased risk of selection bias. For instance, we identified sociodemographic differences between the included and excluded samples, which may reflect previously identified sociodemographic differences in physical health monitoring for people with severe mental illness in primary care.^30^ However, our secondary analyses included larger sample sizes and produced consistent results. Second, weight data recording in primary care may be subject to informative missingness. Individuals who experience rapid weight changes may be monitored more closely than those who have a stable weight. This may bias our included sample towards more extreme weight changes. Additionally, our results regarding weight changes may in part reflect the statistical phenomenon of regression to the mean, rather than being biologically driven. Third, we were unable to distinguish between intentional and unintentional weight loss. However, given the prodromal context, we consider that the majority of observed weight loss prior to FEP is most likely to be unintentional. Fourth, the recorded primary care FEP date may not exactly match psychosis onset due to delays in help-seeking and/or delays in diagnoses being recorded in primary care. We accounted for this with our 6-month pre-FEP index window. However, this meant that we could not account for the time between antipsychotic initiation and the measurement of weight. Fifth, while we addressed a number of potential confounders, residual confounding remains likely. For instance, the presence of negative symptoms leading to poor self-care as well as severity of positive symptoms could contribute to more extreme weight change through health behavioural mechanisms and altered antipsychotic doses. Sixth, while we used broad ICD codes for study population ascertainment reflective of the heterogeneous psychosis early intervention population in the UK, accurate ascertainment from routine health records may be limited by diagnostic uncertainty or incomplete/incorrect recording. Seventh, our follow-up period was limited to 18 months following FEP diagnosis, and so we cannot make any inference about longer-term trends. Finally, the observed prevalence of antipsychotic prescription and weight recording post-FEP may be inaccurate and under-reported. Replication of our analysis in linked primary and secondary mental healthcare datasets, when possible, may help to address this.

In conclusion, weight loss before FEP is an under-recognised and clinically relevant feature of the prediagnosis period. In our primary care sample, individuals with pre-FEP weight loss experienced substantially greater and earlier postdiagnosis weight gain, often exceeding the amount of weight lost and this pattern was robust to sensitivity analyses and adjustment included for antipsychotic prescription.

### Clinical implications

Incorporating prediagnosis weight trajectories into clinical assessment may improve early identification of individuals at risk of pronounced weight gain in the initial period following diagnosis. This identification may inform interventions during the initial treatment period and may help avoid consequences of short-term weight gain.

## Supplementary material

10.1136/bmjment-2026-302720 online supplemental file 1

## Data Availability

Data are available upon reasonable request. Data may be obtained from a third party and are not publicly available.
