# Supplementary material for: Weight loss prior to diagnosis of first-episode psychosis predicts subsequent weight gain: a retrospective cohort study of the UK Clinical Practice Research Datalink (CPRD) primary care database
Source: BMJ Ment Health. 2026 Jul 16;29(1):e302720. doi: 10.1136/bmjment-2026-302720 (PMC13384133; doi:10.1136/bmjment-2026-302720)
Supplement: online supplemental file 1 [file bmjment-29-1-s001.docx]

# Supplementary material

## Methods

### Linkage methods

We used person-level linkage for primary care data with Hospital Episode Statistic data, and small-area (practice level) linkage for the generation of Townsend Scores. CPRD primary care records were linked by NHS Digital/NHS England, acting as a trusted third party, using deterministic matching on NHS number, date of birth, sex, and postcode within an 8-stage algorithm.[1] The study team did not undertake record linkage directly. We used CPRD standard linked datasets, for which CPRD provides linkage eligibility and match-quality metadata; these standard linked datasets are restricted to higher-quality matches intended to minimise false matches. For the deprivation measure, practice-level Townsend scores were obtained via CPRD small-area linkage based on practice postcode in England.


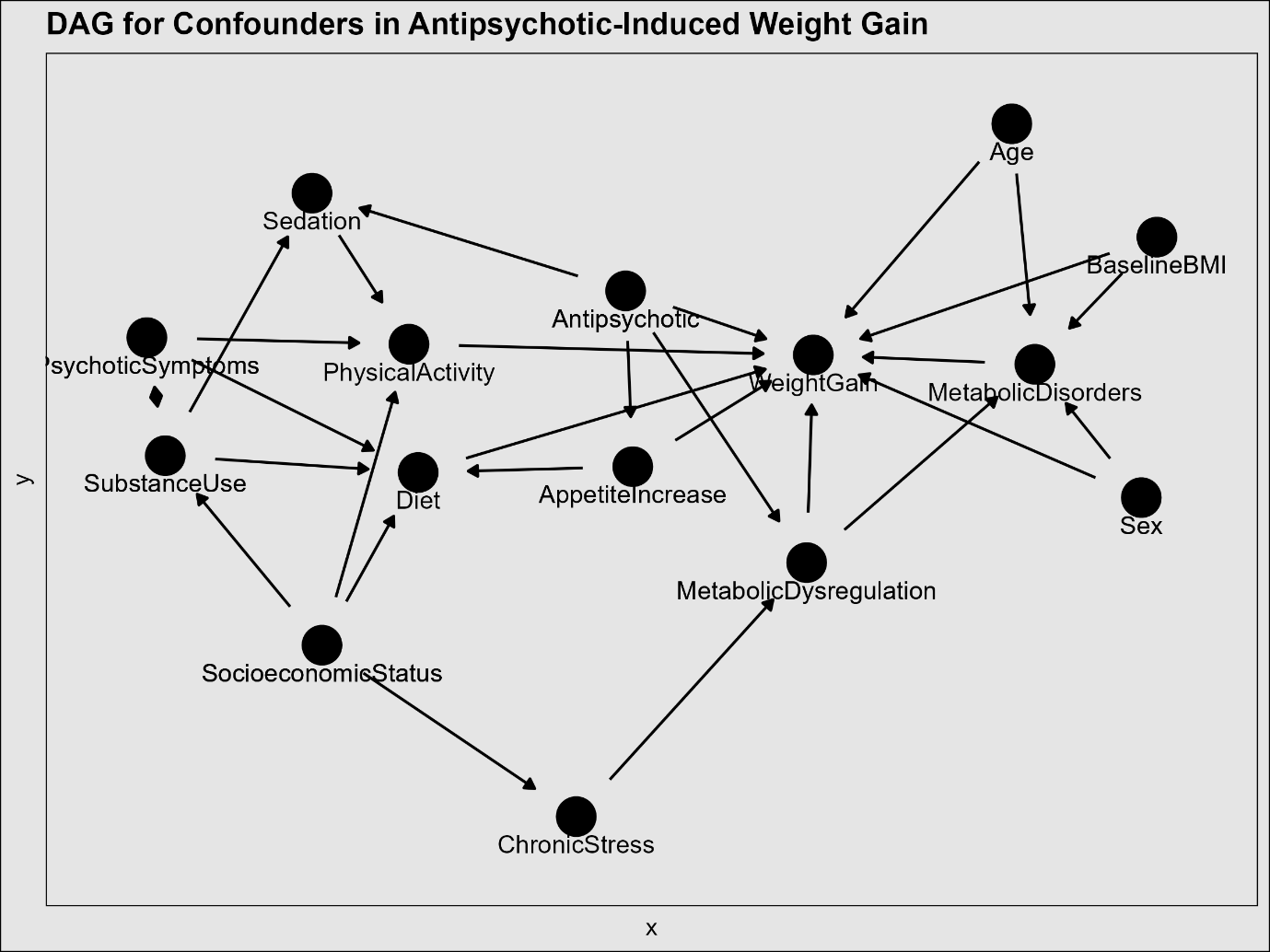

Supplementary figure 1: Directed Acyclic Graph of potential confounders for weight change
Note: We created this figure a priori from previous research and clinical judgement. BOM (First author) created a first draft of the figure, with refinements and suggestions offered by the wider authorship team

### List of categories in covariates

**Sex**: Male, Female

**Age at FEP**: Continuous measure

**Psychiatric Diagnosis**:

- Acute and Transient Psychotic Disorder.
- Delusional Disorder.
- Depression with Psychotic Symptoms.
- Mania with Psychotic Symptoms / Affective Psychosis.
- Organic Psychotic Disorder.
- Postpartum Psychosis.
- Schizoaffective Disorder.
- Schizoid / Schizotypal Personality Disorder.
- Schizophrenia.
- Substance-Induced Psychotic Disorder.
- Unclassified/Other Psychotic Disorder.
- Missing.

**Geographical and Socioeconomic Covariates**

- **Location**:
  - East Midlands.
  - East of England.
  - London.
  - North East.
  - North West.
  - South East.
  - South West.
  - West Midlands.
  - Yorkshire & The Humber.
  - Missing.
- **Ethnic Group**:
  - Black African or Caribbean.
  - East Asian.
  - Mixed.
  - Other.
  - South Asian.
  - White / European.
- **Townsend Quintile (Deprivation Score)**:
  - 1 (Least Deprived).
  - 2.
  - 3.
  - 4.
  - 5 (Most Deprived).
  - Missing.
- **Baseline Antipsychotic**:
  - 0 (No antipsychotic recorded).
  - Specific agents: Amisulpride, Aripiprazole, Asenapine, Chlorpromazine, Clozapine, Flupentixol, Fluphenazine, Haloperidol, Levomepromazine, Olanzapine, Paliperidone, Pericyazine, Pimozide, Pipotiazine, Prochlorperazine, Quetiapine, Risperidone, Sulpiride, Trifluoperazine, Zuclopenthixol.

### BOBYQA (Bound Optimization BY Quadratic Approximation) optimiser

We employed the BOBYQA (Bound Optimization BY Quadratic Approximation) optimiser within the lme4 R package to estimate our longitudinal mixed-effects models. Because our analysis of weight change following first-episode psychosis involved repeated measures with random slopes nested within individuals, we required a robust numerical approach to ensure model convergence. We chose BOBYQA specifically because it effectively handles the multidimensional parameter space of our models by iteratively constructing quadratic approximations of the objective function, which proved essential for achieving stable estimates in the presence of varied pre-FEP weight trajectories.

### Missing data

We conducted multiple imputation using chained equations (MICE) to handle missing data in our covariates, generating 20 imputed datasets to ensure the stability of our estimates. To ensure each variable was handled according to its specific distribution, we applied different statistical models within the imputation process. We used Predictive Mean Matching (PMM) for continuous variables, which maintains the original range of the data by assigning missing values based on observed values from similar participants. For binary variables, such as sex, we employed Logistic Regression to estimate the probability of category membership. For categorical variables with more than two levels, such as ethnicity or diagnosis, we used Polytomous Logistic Regression to account for multiple potential categories. Finally, we pooled the results from all 20 datasets using Rubin’s rules to produce our final adjusted estimates and confidence intervals.

We imputed data for BMI, diagnosis and location, whose levels of missing data were relatively low across all cohorts. BMI missingness was 3.8% in the *strict definition* analysis, 6.3% in the *extended definition* analysis, and 8.8% in the *loosest* definition analysis. Location missingness was 6.2% in the *strict definition* analysis, 5.4% in the *extended definition* analysis, and 6.3% in the *loosest* definition analysis. Psychiatric Diagnosis missingness was 0% in the *strict definition,* 0.1% in the *extended definition*, and 0.3% in the *loosest definition* analysis.

Supplementary table 1 shows the auxiliary variables used in the MICE process


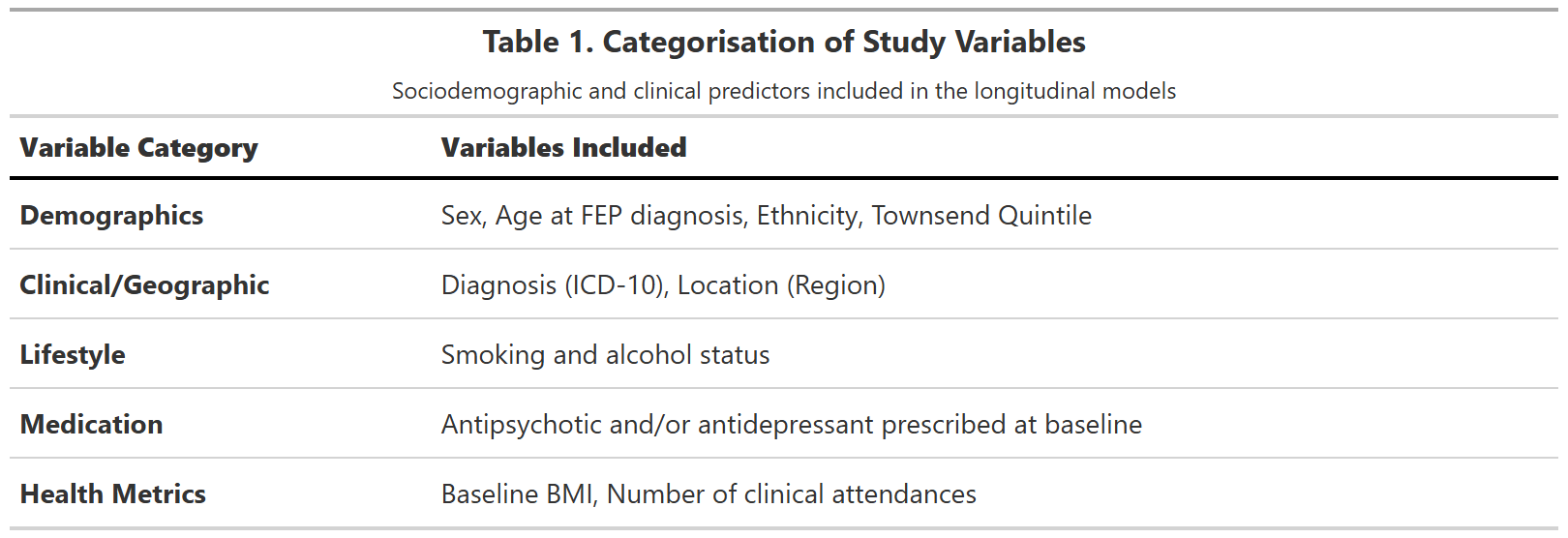

Supplementary table 1: Auxiliary variables for imputation


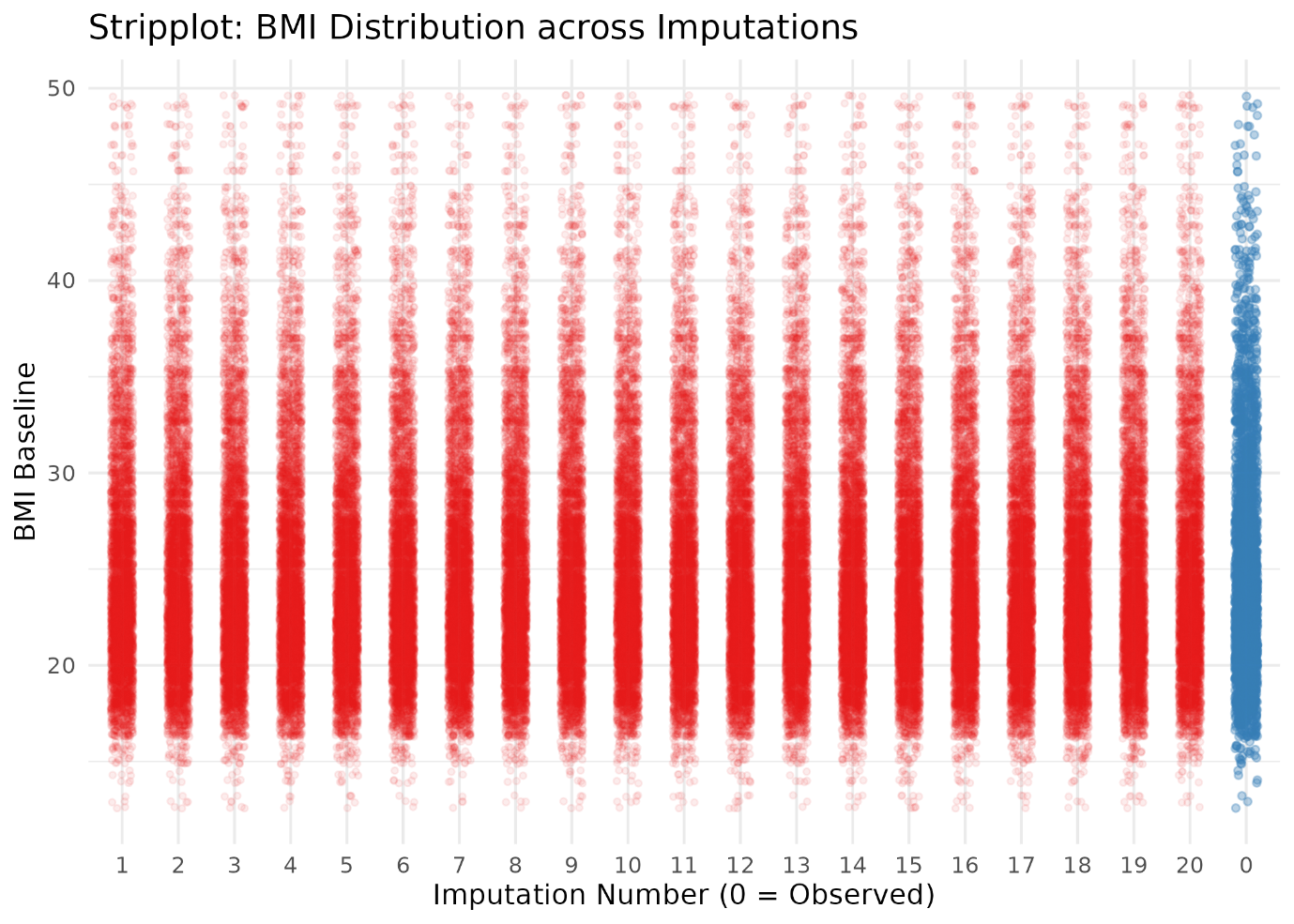

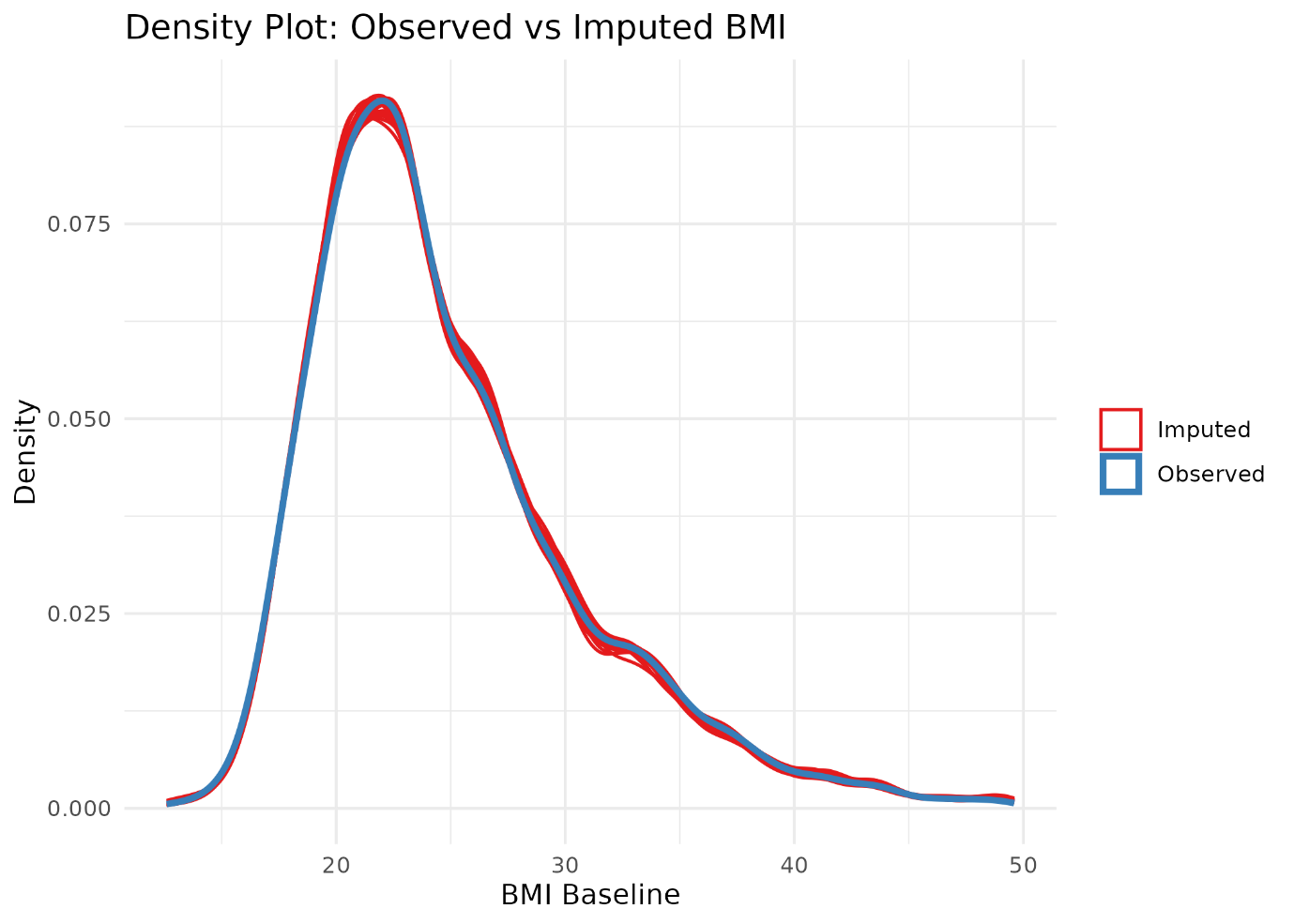


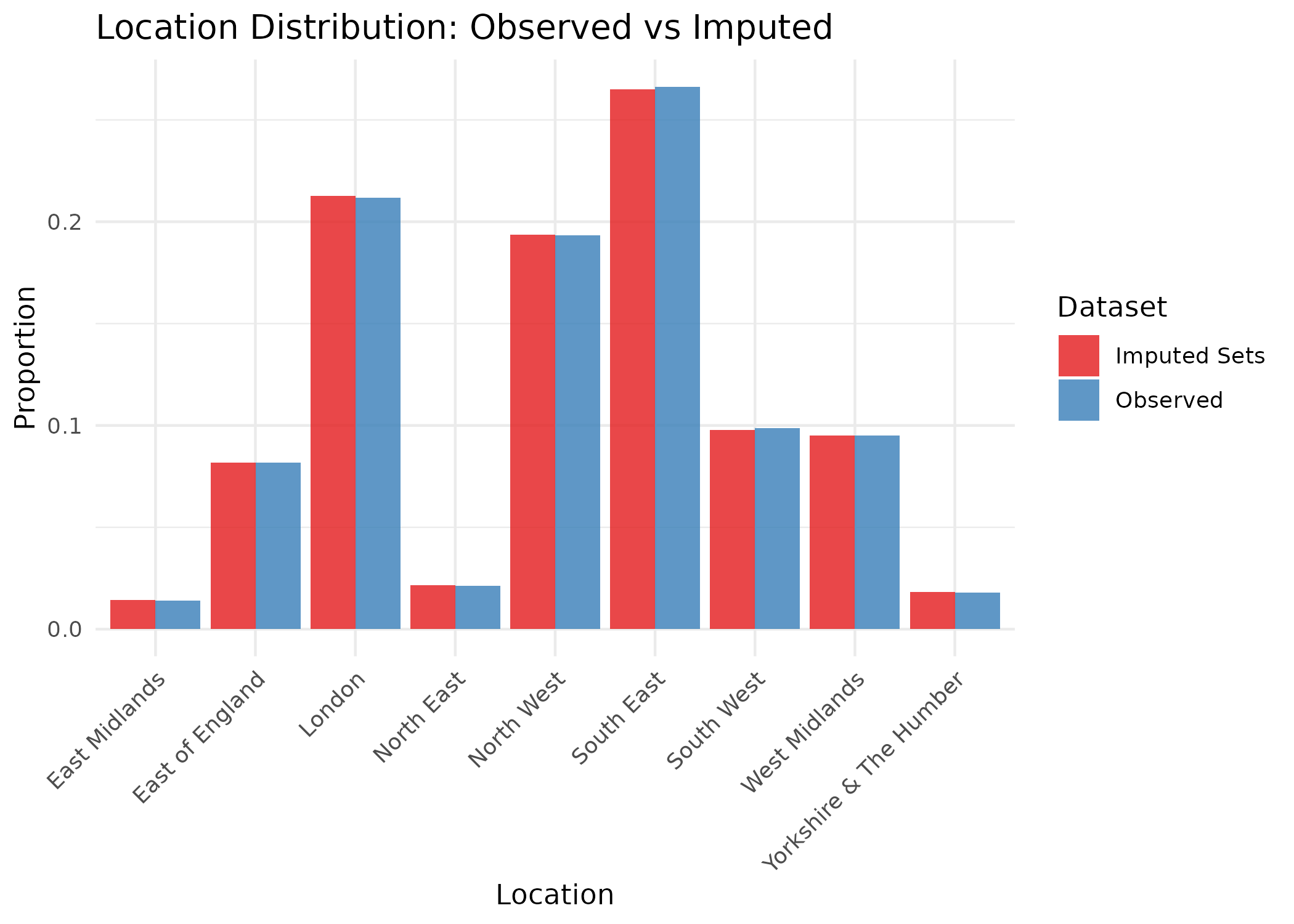


Supplementary figure 1: Imputation results

## Distribution of weight changes by timepoint

| **Time period** | **Count** | **Mean weight change** | **SD weight change** | **Skewness** | **Kurtosis** |
| --- | --- | --- | --- | --- | --- |
| **(365,-304] days** | 44 | 0.0019 | 0.113 | 0.199 | 1.072 |
| **(-304,-244] days** | 108 | -0.0126 | 0.093 | -0.7 | 7.89 |
| **(-244,-183] days** | 149 | 0.0113 | 0.079 | 0.474 | 4.187 |
| **(-183, -122] days** | 164 | 0.0039 | 0.084 | 0.768 | 2.398 |
| **(-122, -60.9] days** | 155 | 0.0022 | 0.078 | 0.317 | 2.392 |
| **(-60, 0] days** | 83 | -0.0026 | 0.106 | 0.24 | 0.846 |
| **(0, 14] days** | **80** | **0.052** | **0.105** | **2.706** | **8.936** |
| **(14, 28] days** | 84 | 0.0131 | 0.076 | -0.264 | 11.891 |
| **(28, 56] days** | 194 | 0.0204 | 0.0902 | -0.077 | 8.626 |
| **(56, 72] days** | 101 | 0.0143 | 0.0743 | 0.7 | 11.955 |
| **(72, 128] days** | 349 | 0.0161 | 0.0664 | 0.181 | 12.61 |
| **(128, 365] days** | 1137 | 0.0107 | 0.0694 | -0.358 | 8.158 |

**Supplementary table 2:** Numerical summaries of the distribution of individuals weight change from one timepoint to the next, split by days from the index diagnosis.

## Results

**Supplementary table 3** shows the characteristics of individuals included in the main analysis, compared to those who were not

| **Characteristic** | **Excluded from Analysis (n = 18,214)** | **Included in Analysis (n = 369)** | **P** |
| --- | --- | --- | --- |
| **Female** | 9069 (49.8) | 245 (66.4) | <0.001 |
| **Age at FEP onset: Mean (SD)** | 26.46 (5.39) | 26.02 (5.98) | 0.750 |
| **Diagnosis** | **N (%)** | **N (%)** | <0.001 |
| Acute and Transient Psychotic Disorder | 1142 (6.3) | 21 (5.7) |  |
| Delusional Disorder | 711 (3.9) | 10 |  |
| Depression with Psychotic Symptoms | 1117 (6.1) | 44 (11.9) |  |
| Mania with Psychotic Symptoms | 5753 (31.6) | 78 (21.1) |  |
| Organic Psychotic Disorder | 81 (0.4) | * |  |
| Postpartum Psychosis | 196 (1.1) | * |  |
| Schizoaffective Disorder | 417 (2.3) | 10 |  |
| Schizoid / Schizotypal Personality Disorder | 97 (0.5) | * |  |
| Schizophrenia | 3387 (18.6) | 19 (5.1) |  |
| Substance-Induced Psychotic Disorder | 194 (1.1) | * |  |
| Unclassified/Other Psychotic Disorder | 5103 (28.0) | 177 (48.0) |  |
| Missing | 16 (0.1) | 0 (0.0) |  |
| **Location** | **N (%)** | **N (%)** | 0.05 |
| East Midlands | 231 (1.3) | 10 |  |
| East of England | 1338 (7.3) | 22 (6.0) |  |
| London | 3192 (17.5) | 61 (16.5) |  |
| Northeast | 287 (1.6) | 10 |  |
| Northwest | 3536 (19.4) | 83 (22.5) |  |
| Southeast | 4890 (26.8) | 94 (25.5) |  |
| Southwest | 1963 (10.8) | 27 (7.3) |  |
| West Midlands | 1748 (9.6) | 32 (8.7) |  |
| Yorkshire & The Humber | 288 (1.6) | * |  |
| Missing | 741 (4.1) | 23 (6.2) |  |
| **Ethnicity** | **N (%)** | **N (%)** | 0.001 |
| Black African or Caribbean | 1129 (6.2) | 33 (8.9) |  |
| East Asian | 296 (1.6) | 10 (2.7) |  |
| Mixed | 3236 (17.8) | 95 (25.7) |  |
| Other | 439 (2.4) | 7 (1.9) |  |
| South Asian | 634 (3.5) | 20 (5.4) |  |
| White / European | 12480 (68.5) | 204 (55.3) |  |
| **Townsend Quintile** | **N (%)** | **N (%)** | 0.167 |
| 1 | 1842 (10.1) | 31 (8.4) |  |
| 2 | 2435 (13.4) | 44 (11.9) |  |
| 3 | 3268 (17.9) | 66 (17.9) |  |
| 4 | 5009 (27.5) | 86 (23.3) |  |
| 5 | 5561 (30.5) | 142 (38.5) |  |
| Missing | 99 (0.5) | 0 (0.0) |  |
| **Baseline BMI** |  |  |  |
| Mean (SD) | 25.63 (6.18) | 25.73 (6.84) | 0.774 |
| **Recorded antipsychotic prescription** | 6948 (38.2%) | 236 (64%) | <0.001 |

Supplementary table 3: Characteristics of those included in the primary analysis vs those who were not
Note: Cells with counts less than 15 have been altered in conjunction with NHS digital reporting guidelines

## Sensitivity analyses

### Differing datasets

**Extended index sample**

In the model using weight loss as a binary variable, those who lost weight were expected to gain 7.94% per year (95% CI 5.62% to 10.27%) of their body weight vs 2.55% per year (95% CI 0.15% to 4.96%) in those who did not lose weight, a difference of 5.39% per year (p = 0.0016). In the model using weight loss as a continuous variable, every 1% loss in body weight was associated with a 0.20% extra weight gain per year (95% CI 0.04 to 0.35, p = 0.011).

**Loose definition sample**

In the model using weight loss as a binary variable, those who lost weight were expected to gain 8.42% per year (95% CI 6.28% to 10.55%) of their body weight vs 1.50% per year (95% CI -0.28% to 3.29%) ^2^in those who did not lose weight, a difference of 6.91% per year (p < 0.0001). In the model using weight loss as a continuous variable, every 1% loss in body weight was associated with a 0.42% extra weight gain per year (95% CI 0.39 to 0.45, p < 0.001).

### Differing adjustments

**Adjustment for Time from Maximum Weight**

When additionally adjusting for the time elapsed between a person’s maximum weight and their index episode, the results remained consistent with the primary analysis. In the binary model, those who lost weight were expected to gain 8.31% per year (95% CI 6.34% to 10.29%), while those who did not lose weight gained 4.16% less per year (interaction estimate 4.15, p = 0.005). In the continuous model, every 1% of body weight loss prior to the index date was associated with a 0.53% extra weight gain in the following 12 months (interaction estimate -0.32 per year, p = 0.010).

**Sociodemographic Adjustment Only**

In a separate model adjusting only for sociodemographic factors (including sex, age, ethnicity, and Townsend deprivation index), the association between pre-index weight loss and post-index gain persisted. For the binary variable, those with pre-index weight loss were expected to gain 8.22% per year (95% CI 6.18% to 10.26%), which was 4.09% per year more than those without weight loss (p = 0.008). The continuous model showed that every 1% of pre-index weight loss led to an extra 0.32% weight gain per year (95% CI 0.14% to 0.50%, p < 0.001).

**Stratification by sex**

Female patients who lost weight pre-diagnosis exhibited a significantly more rapid weight gain trajectory, with adjusted models showing an 8.8% (95%CI 6.43 to 11.20) annual increase compared to just 3.7% (95%CI 0.95 to 6.38) for those who maintained their weight, a difference of 5.05% per year (95%CI of 1.47 to 8.63%). Meanwhile, male patients who lost weight pre-diagnosis gained 6.52% body weight per year (95%CI 2.49 to 10.55%) compared to 3.98% per year (95%CI 0.15 to 7.81%), a difference of 2.54% (-3.02 to 8.1%). The continuous analysis gave similar results for both groups, with each percentage of body weight lost expected to lead to 0.3% additional weight gain in females (95% CI 0.09 to 0.51%) and 0.33% additional weight gain in males (95%CI 0.05 to 0.61%).

### Differing models

**Rate of Weight Change**

When the independent variable was changed to the rate of weight change (percentage change per year), every 1 unit increase in the rate of pre-index weight loss was associated with an extra 0.17% weight gain per year post-index (95% CI 0.12% to 0.22%, p < 0.001). In the binary version of this model, the difference between those who lost weight and those who did not was 4.15% per year (interaction estimate 4.15, p = 0.005).

**Raw Weight Change**

Using raw weight change (in kilograms) as the predictor, every 1 kg of weight loss prior to the index episode was associated with an extra 0.25% annual weight gain following the start of treatment (95% CI 0.12% to 0.37%, p < 0.001). In the binary model, the expected annual gain for those who lost weight was 5.37% (95% CI 3.93% to 6.81%), which was 2.81% higher than the gain observed in those who did not lose weight (p = 0.010).

**Zero intercept model**

For this model, we constrained the intercept to zero, so as to reflect the fact that, by definition, a person’s weight change at time 0 must be zero. In this model using weight loss as a binary variable, those who lost weight were expected to gain 8.23% per year (95% CI 6.15% to 10.31%) of their body weight vs 4.07% per year (95%CI 1.82% to 6.32%) in those who did not lose weight, a difference of 4.16% per year (95%CI 1.92% to 7.80%, p = 0.001). In the model using weight loss as a continuous variable, every 1% loss in body weight was associated with a 0.41% extra weight gain per year (95% CI 0.23 to 0.59, p < 0.001).

The effect of pre-index weight loss on post-index weight gain was larger over time periods closer to the index date. Every 1% of weight loss prior to diagnosis was expected to lead to an extra 0.62% weight gain (95% CI 0.4% to 0.84%) in the following 12 months and an extra 0.8% weight gain per year in the following 6 months (equivalent to an extra 0.4% in the first 6 months).

A summary of the sensitivity analyses is presented in **Supplementary Table 4:**


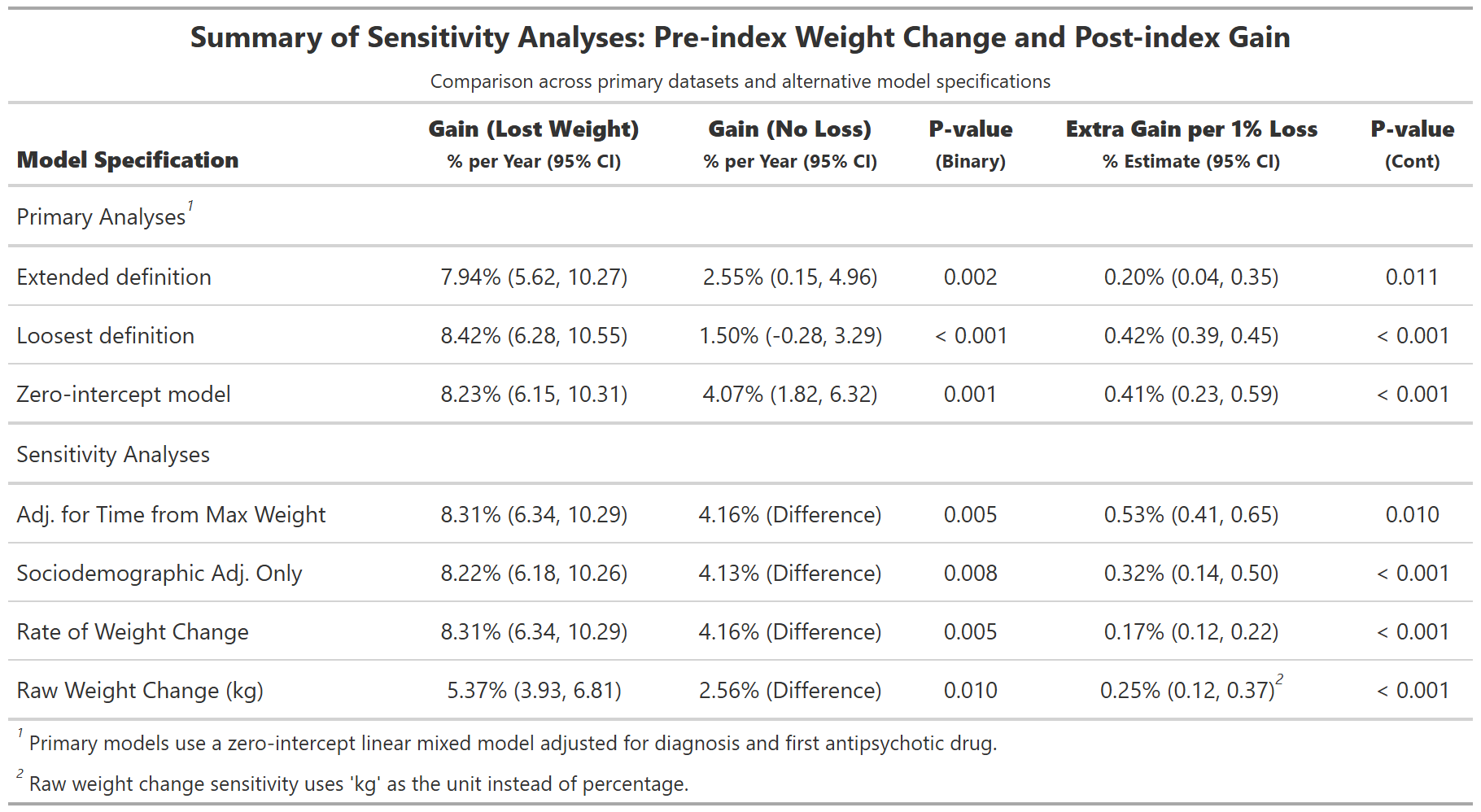

Supplementary Table 4: Summary of sensitivity analyses

Supplementary table 5 gives a summary of the results of the primary analysis

| **Primary Analysis: Impact of Pre-index Weight Loss on Post-index Gain^2^** | | | | |
| --- | --- | --- | --- | --- |
| *Linear mixed models for body weight trajectory* | | | | |
| **Analysis Focus** | **Unadjusted Estimate (95% CI)** | **P-value** | **Adjusted Estimate (95% CI)^1^** | **P-value** |
| Binary: Difference in Annual Gain (Lost vs No Loss) | 4.86% (1.92, 7.80) | 0.001 | 4.15% (1.10, 7.22) | 0.005 |
| Continuous: Extra Annual Gain per 1% Pre-index Loss | 0.41% (0.23, 0.59) | < 0.001 | 0.32% (0.14, 0.49) | < 0.001 |
| Time-limited: Extra Annual Gain (First 12 Months) | 0.47% (0.25, 0.69) | < 0.001 | 0.48% (0.27, 0.69) | < 0.001 |
| Time-limited: Extra Annual Gain (First 6 Months) | 0.62% (0.35, 0.89) | < 0.001 | 0.60% (0.32, 0.88) | < 0.001 |
| 1 Adjusted for age, sex, BMI at index, diagnosis, antipsychotic drug, socioeconomic status, ethnicity, location, and GP visit frequency. | | | | |

Supplementary table 5: Summary of primary analyses

1. Wolf A, Dedman D, Campbell J, Booth H, Lunn D, Chapman J, et al. Data resource profile: clinical practice research datalink (CPRD) aurum. International journal of epidemiology. 2019;48(6):1740–g.
